# CT‐Based Radiostereometric Analysis: A Promising Alternative to Conventional Radiostereometric Analysis in Determining Polyethylene Wear in Total Hip Arthroplasty

**DOI:** 10.1155/aort/8544470

**Published:** 2026-09-16

**Authors:** Elin Kramer, Olof Sandberg, Anders Brüggemann, Nils P. Hailer, Olof Sköldenberg, Cyrus Brodén

**Affiliations:** ^1^ Section of Orthopedics and Hand Surgery, Department of Surgical Sciences, Uppsala University, Uppsala, Sweden, uu.se; ^2^ Sectra AB, Linköping, Sweden; ^3^ Division of Orthopedics, Department of Clinical Sciences, Danderyd Hospital, Karolinska Institutet, Stockholm, Sweden, ki.se

**Keywords:** CT-RSA, RSA, total hip arthroplasty, wear

## Abstract

**Background:**

CT‐based radiostereometric analysis (CT‐RSA) enables the measurement of early implant migration after Total Hip Arthroplasty (THA) with high precision and accuracy at a low effective radiation dose. This study is the first to investigate whether CT‐RSA might be comparable to conventional radiostereometric analysis(RSA) even for in vivo polyethylene liner wear measurements after THA.

**Purpose:**

To evaluate the comparability of CT‐based RSA and conventional RSA for measuring polyethylene liner wear after THA.

**Materials and Methods:**

A total of 8 patients treated with a reverse hybrid THA (cemented cup and uncemented stem) were included from an ongoing RSA study. CT‐RSA and RSA images were taken immediately postoperatively and after 6 years to compare measurements of the polyethylene wear. Double examinations were performed to estimate the precision of the CT‐RSA method.

**Results:**

Wear measured as median proximal translation of the femoral head into the cup at 6 years was 0.10 mm (IQR 0.02–0.17 mm) as measured by RSA and 0.13 mm (IQR 0.04–0.27 mm) by CT‐RSA. Bland–Altman plots indicated good agreement between the methods; for proximal wear the mean difference between methods was 0.04 mm (limits of agreement −0.37–0.46 mm). Proximal wear precision was 0.16 mm for RSA and 0.15 mm for CT‐RSA. The mean effective radiation dose was 0.2 mSv per CT scan and 0.04 mSv per RSA scan.

**Conclusion:**

This exploratory study suggests that CT‐RSA provides wear measurements comparable to those obtained with conventional RSA and may therefore represent a viable alternative for evaluating liner wear in future implant studies.

**Trial Registration:** ClinicalTrials.gov: NCT02254980

## 1. Introduction

Abrasive wear of the polyethylene liner is a common cause of aseptic loosening after Total Hip Arthroplasty (THA). The erosion of the polyethylene liner generates debris, triggering an inflammatory response and subsequent osteolysis and loosening [1]. Efforts have been undertaken to reduce wear by developing liners with antioxidant effects, for example, Vitamin E‐infused highly cross‐linked polyethylene (VEPE) liners, or the use of a gamma inert gas sterilization process to obtain a higher degree of cross‐linking [2].

Radiostereometric analysis (RSA) has served as the gold standard for detecting migration and wear of orthopedic implants since the 1980s [3]. However, conventional RSA comes with inherent limitations, such as the requirement for markers, specialized hardware, laboratories, and qualified personnel. Recently, Computed Tomography‐based RSA (CT‐RSA) has emerged as a promising alternative for migration measurements, as it overcomes some of the limitations of conventional RSA. It has so far demonstrated comparable precision and accuracy to conventional RSA for early implant migration measurements [4, 5]. A recent study by Polus et al. [6] found that CT‐RSA demonstrates excellent precision for wear measurements in an experimental setting, regardless of head size, head type, or low radiation dose. However, no study has yet explored whether CT‐RSA can measure liner wear in vivo with a comparable precision to RSA.

The aim of this study was thus to test the agreement between CT‐RSA and conventional RSA for in vivo measurements of wear of cemented polyethylene cups at 6 years postoperatively. Furthermore, we intended to assess the precision of CT‐RSA for wear measurements.

## 2. Materials and Methods

This exploratory study included 8 patients with valid RSA wear measurements from the last 11 consecutive patients from an ongoing 6‐year follow‐up randomized controlled study (RCT) conducted at Danderyd Hospital between 2013 and 2015 [7]. All patients included in the study provided written informed consent to participate. In that RCT, patients with hip osteoarthritis eligible for primary reverse hybrid THA were randomized to receive either a standard UHMWPE liner, argon‐gas‐gamma‐irradiated at 100 kGy (ArCom, Zimmer Biomet, Warsaw, IN), or a UHMWPE liner infused with Vitamin E, also argon‐gas‐gamma‐irradiated at 100 kGy (E1, Zimmer Biomet, Warsaw, IN). Details about inclusion and exclusion criteria for the original study population can be found in the published study protocol [8]. An amendment to the original ethics approval was obtained to allow CT scans to be performed for the last 11 patients included in the RCT for the purposes of this study. Of these 11 patients, 3 were excluded due to insufficient quality of the RSA measurements (CN > 150, ME > 0.35) to allow comparison between RSA and CT‐RSA. In the sub‐population of eight included in this study, six patients received a VEPE liner and two a standard PE liner (Table 1).

**TABLE 1:** Baseline characteristics of the patients in the standard PE and VEPE groups at time of surgery.

Patient	Age (years)	Sex (female male)	ASA PS classification^∗^	Type of liner	Body mass index (BMI)
1	65	F	3‐4	VEPE	37.2
2	58	F	1‐2	VEPE	20.9
3	71	F	1‐2	VEPE	26.3
4	75	M	1‐2	VEPE	20.7
5	65	M	1‐2	PE	24.2
6	66	M	3‐4	VEPE	29.7
7	63	F	1‐2	PE	25.5
8	59	M	1‐2	VEPE	27.5

^∗^The American Society of Anesthesiologists (ASA) Physical Status Classification System is used to categorize a patient’s preoperative overall health, with higher classes indicating greater systemic disease burden and increased perioperative risk.

The acetabular component comprised the Exceed ABT cemented cup (Zimmer‐Biomet, Warsaw, IN) with metallic wire encircling the cup, while the stem consisted of an uncemented Bi‐Metric, tapered, proximally porous, hydroxyapatite‐coated titanium alloy with a 32 mm cobalt‐chromium head (Zimmer‐Biomet, Warsaw, IN). Nine tantalum beads (with a diameter of 1.0 mm) were surgically inserted into the acetabular bone around the cup and another nine within the cup prior to cementation for subsequent RSA measurements. Postoperatively, full weight‐bearing was permitted with no restrictions on range of motion. These patients underwent RSA examinations at the second postoperative day, 3 months, and 1‐, 2‐, and 6‐years following surgery to measure cup migration and polyethylene wear. CT examinations were taken postoperatively and at 6 years following surgery in order to measure wear by CT‐RSA and compare the results to measurements obtained by conventional RSA. The CT scans were taken on the same day as the RSA examinations after the same amount of weight‐bearing.

The study protocol for the original RCT has been previously published elsewhere [8].

### 2.1. Conventional RSA Method

A digital radiograph and uniplanar RSA calibration cage (Cage 43, RSA Biomedical AB, Umeå, Sweden) were used to acquire the RSA images. A flat panel digital detector (Bucky Diagnostics Philips, Eindhoven, the Netherlands) was used to acquire the radiographic images. UmRSA 6.0 computer software (RSA Biomedical, Umeå, Sweden) was used for all RSA migration analyses. The built‐in edge‐detection tool in the software was used to determine head penetration into the liner. The analysis was performed by an experienced RSA user from UmRSA who was blinded to what type of polyethylene was used in each patient. All patients were included in whom the condition numbers (CN) for the tantalum beads were under 150 and who also had a mean error (ME) of rigid body fitting below 0.35 mm, indicating good spread and stability of the beads [9]. The wear was presented as orthogonal translation (along *x*‐, *y*‐, and *z*‐axes) and maximum total point motion (MTPM) for the center of the head in relation to the beads in the acetabular component. Precision measurements for RSA were conducted at 12 months following surgery and were performed on the same day with the patient being repositioned on the table between the examinations.

### 2.2. CT‐RSA Method

To acquire CT images, a 128‐detector CT scanner was used (Discovery CT750HD, GE Healthcare, Chicago, IL, USA). The CT protocol was set to 120 kV, 10 mAs, slice thickness 0.625 mm, increment of 0.312 mm, rotation time 1 s, Pitch 0.98, and metal artifact reduction was used for all scans. The CT scans were analyzed with the CT‐RSA image registration software CTMA (Version 25.2, Sectra, Linköping, Sweden). For precision measurements, 2 consecutive scans were performed on the same day with the patient being repositioned on the table between the scans. The double examinations were performed directly postoperatively or for one patient at 3 months following surgery.

**FIGURE 1:**
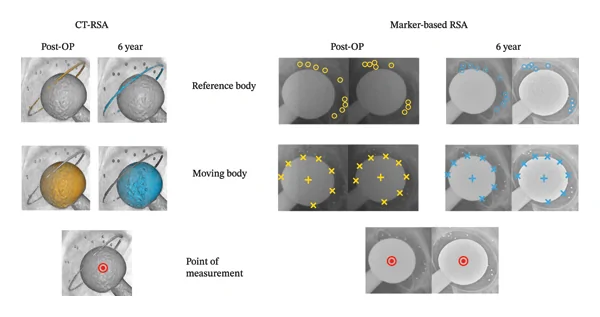
On the left, an illustration depicts the initial steps of the CT‐RSA workflow prior to alignment. The reference body (thread of the cup) and moving body (head of stem) have been detected, with the measurement point set at the center of the head. On the right, the corresponding steps for standard RSA are shown.

In this study, the following steps in the CT‐RSA process were conducted. Firstly, the two CT volumes were imported into the CT‐RSA software. A segmentation was then performed manually for each patient to visualize both the metallic thread in the acetabular cup and the prosthetic head (threshold value aimed at 2200 Hounsfield Units). Secondly, the reference body (the thread of the cup, or, in one patient in whom the thread was missing, the tantalum beads inside the cup) was defined from baseline and follow‐up CT examinations. The two threads from the baseline and follow‐up examinations were aligned and matched using landmark‐based computer‐assisted merging, which ensured accurate matching of the cup position across the two different time points (Figure 1). Similarly, the moving rigid body (the prosthetic head) was defined, aligned, and matched in the same manner from the baseline and follow‐up examinations. Finally, the coordinate system was adjusted, and a point of measurement (center of head) was defined. The CTMA software calculated the movement of the prosthetic head compared to the metallic thread of the cup between the two different timepoints of the CT scans.

**FIGURE 2:**
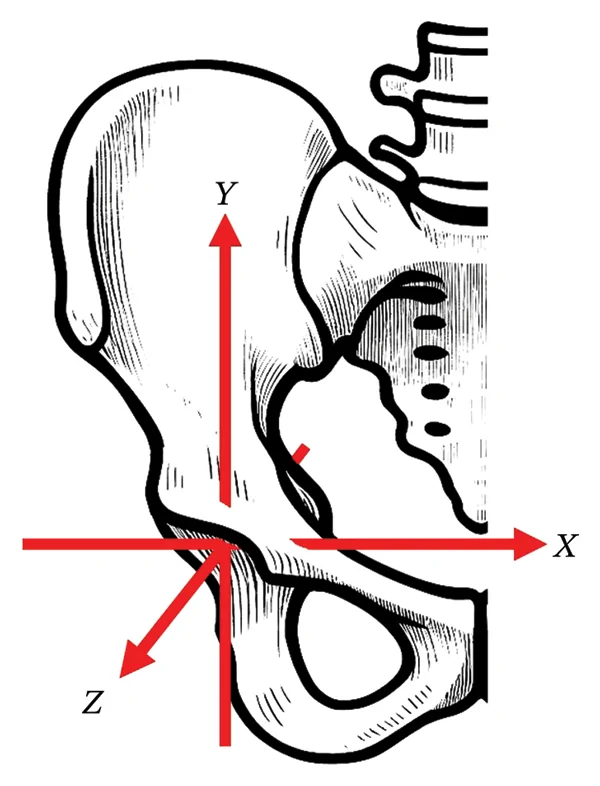
The coordinate system and the direction for measuring movement along the different axes. Hence, positive values along *x*‐axis are considered medial translation, positive values along the *y*‐axis proximal translation, and positive values along the *z*‐axis anterior translation.

The coordinate system used for the CT‐RSA images in this study (Figure 2) was adapted to the standard RSA coordinate system to ensure that the wear values were comparable. Positive values along the *x*‐axis indicate medial translation, positive values along the *y*‐axis proximal translation, and positive values along the *z*‐axis anterior translation of the prosthetic head. In accordance with CT‐RSA guidelines [9], we chose to report total translation (TT) rather than MTPM. For femoral head migration, TT and MTPM are calculated identically. TT is the total distance travelled for a given point regardless of direction and is calculated using Pythagoras’ theorem. Translation was reported for the center of the prosthetic head, while rotation was set to zero due to the measure of wear and not migration. No RSA examinations were excluded due to marker occlusion. One of the cups lacked a metal thread suitable for CT‐RSA analysis, so tantalum beads inserted in the cup were used instead.

### 2.3. Effective Radiation Dose

The CT effective dose was calculated using the dose length product (DLP) multiplied by a pelvic conversion factor: 0.0129 mSv/mGy.cm from IRCP publication 103 [10]. The RSA effective dose was calculated with a Monte Carlo simulation using software PCXMC Dose Calculation available at Danderyd Hospital [11].

### 2.4. Statistics

Due to the small sample size, we employed descriptive statistics for the comparison of the methods using calculations for the median, lower, and upper quartile for the wear measurements since the data were not normally distributed. Bland–Altman plots were used to assess agreement for wear measurements between RSA and CT‐RSA as well as for CT‐RSA measurements between the two observers (EK, OSA). The limits of agreement (LoA) were calculated as mean difference ± *T*‐value (2.365; based on 7 degrees of freedom) × standard deviation (SD).

Precision refers to the closeness of agreement between test results obtained under unchanged conditions (ISO 5725‐1:2023) and is calculated using the ISO standard, multiplying the SD with 1.96 to give the 95% Reference Interval (95% RI). In our cohort, in accordance with CT‐RSA guidelines, precision for CT‐RSA was calculated from double measurements according to the ISO standard, but using a modified *T*‐value of 2.365, based on the above‐described degrees of freedom [12]. Precision for RSA was calculated on a subset of 11 patients from the original RCT rather than the cohort included in the present study (in whom double RSA examinations were not available) using the same approach with a *T*‐value of 2.228 (based on 10 degrees of freedom). The precision ratio (CT‐RSA/RSA) was calculated to quantify the relative precision of the two methods. A ratio below 1 indicates higher precision for CT‐RSA, whereas a ratio above 1 indicates higher precision for RSA. Uncertainty around the ratio was estimated using nonparametric bootstrap resampling (10,000 replicates) to obtain a 95% confidence interval.

A sensitivity analysis was performed by recalculating wear after excluding the patient in whom tantalum beads were used instead of the thread of the cup due to the absence of visible thread. All statistics were performed using R‐statistical software (Version 4.4.1, R Core Team 2024. R: A language and environment for statistical computing. R Foundation for Statistical Computing, Vienna, Austria. URL https://www.R-project.org/).

### 2.5. Outcome Measurements

The primary endpoint was comparison of measurements between CT‐RSA and RSA for proximal migration of the head into the cup (wear along the *y*‐axis; Figure 2) at 6 years follow‐up as an indication for polyethylene wear. The secondary outcome was to estimate precision for CT‐RSA for wear measurements.

### 2.6. Ethics Registration

The original study was approved by the ethics committee of Karolinska Institute (No. 2011/2003‐31/1).

## 3. Results

### 3.1. Wear Measurements at 6 Years and Agreement Between the Methods

**FIGURE 3:**
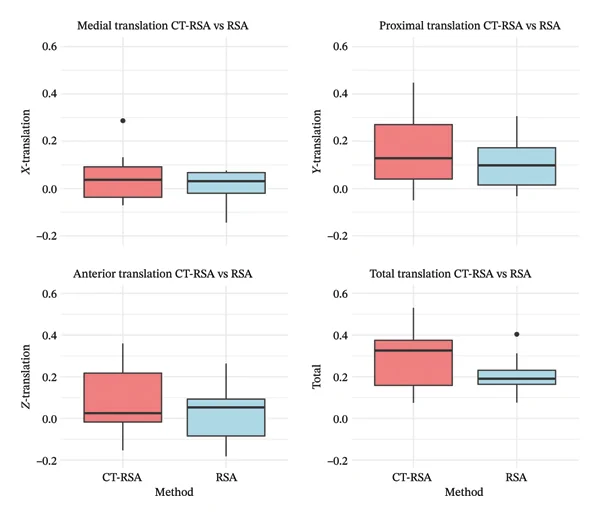
Translation of the center of the prosthetic head at 6 years postoperatively as assessed via CT‐RSA (red) and RSA (blue) in the orthogonal axis and total translation (maximum total point motion for RSA).

The median proximal translation (i.e., wear along the *y*‐axis) of the femoral head into the cup, thus proximal wear at 6 years measured with RSA, was 0.10 mm (IQR 0.02–0.17 mm). For CT‐RSA the corresponding measurement was 0.13 mm (IQR 0.04–0.27 mm; Figure 3).

The median TT of the femoral head into the cup, thus total wear at 6 years measured with RSA, was 0.19 mm (IQR 0.16‐0.23 mm). For CT‐RSA the corresponding measurement was 0.33 mm (IQR 0.16‐0.38 mm; Figure 3).

After exclusion of the patient in whom tantalum beads were used instead of the thread of the cup, the results remained similar.

**FIGURE 4:**
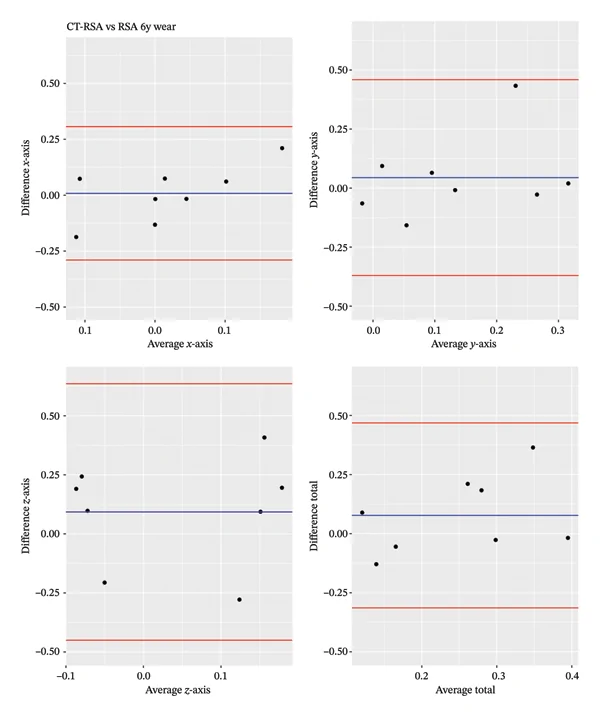
Agreement between CT‐RSA and RSA for wear measurements in *x*, *y*, and *z* translations and total translation (MTPM for RSA) at 6 years postoperatively. The blue line represents the mean difference, and the red lines represent the upper and lower limits of agreement (mean difference ± 2.365 × SD).

**FIGURE 5:**
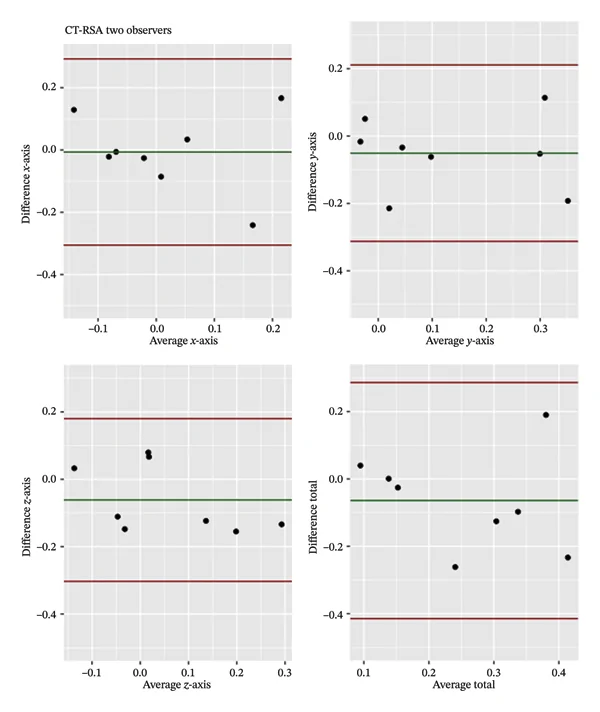
Inter‐rater agreement for wear measurements in *x*, *y*, *z* translations and total translation between two independent observers using CT‐RSA. The green line represents the mean difference and the red lines represent the upper and lower limits of agreement (mean difference ± 2.365 × SD).

Comparison between CT‐RSA and RSA for wear along the *y*‐axis, using Bland–Altman plots, showed that the mean difference was 0.04 mm, with the LoA ranging from −0.37 to 0.46 mm, indicating agreement between methods. Most data points were located within the LoA surrounding the mean difference. Visual inspection of the Bland–Altman plots was used to assess potential bias, and no apparent relationship between the differences and the mean values was observed, suggesting the absence of proportional bias. For further details about wear along the axes, see Figure 4. Interobserver agreement assessed using Bland–Altman analysis demonstrated good agreement between the two observers for CT‐RSA measurements (Figure 5).

### 3.2. Precision for CT‐RSA

The precision for proximal translation of the femoral head into the cup was 0.16 mm (95% RI) for RSA and 0.15 mm (95% RI) for CT‐RSA. The precision ratio (CT‐RSA/RSA) was 0.94 (bootstrap 95% CI 0.47–1.66).

### 3.3. Mean Effective Radiation Dose

The mean effective radiation dose of the examinations used for CT‐RSA was estimated to be 0.2 mSv (range 0.10–0.22). The RSA mean effective dose was estimated to be 0.04 mSv (range 0.036–0.044).

## 4. Discussion

### 4.1. Agreement Between CT‐RSA and RSA

In this exploratory study, we showed that CT‐RSA can be a reliable alternative to conventional RSA for wear measurements with a precision of 0.15 mm. The Bland–Altman analysis indicated good agreement between the two methods, with a mean difference of 0.04 mm for wear along the *y*‐axis. The LoAs were narrower for the y‐ and *x*‐axes compared to the *z*‐axis, indicating better agreement between CT‐RSA and RSA in these directions.

There seems to be an association between a higher degree of wear and later revision due to aseptic loosening. One study implied that the survival rate at 20 years was below 30% if the liner wear rate was above 0.2 mm per year [13]. There also seems to be an association between increased wear above 0.1 mm per year and development of osteolysis which could lead to loosening of the implant [14]. In a review by Dumbleton et al. [15], a practical threshold of 0.05 mm/year was proposed to minimize the risk of osteolysis due to particle‐induced disease. Notably, most of the referenced studies were conducted on earlier generations of polyethylene liners. Since then, the introduction of highly cross‐linked polyethylene (HXLPE) liners has demonstrated excellent long‐term outcomes, with wear rates typically around 0.02 mm/year [16].

The Bland–Altman analysis demonstrated a mean difference of 0.04 mm, indicating minimal systematic bias between CT‐RSA and conventional RSA. Most studies report no increased risk of implant failure and osteolysis when wear remains below 0.2 mm per year [13, 14, 16], corresponding to approximately 1.2 mm over 6 years. The observed LoA between CT‐RSA and conventional RSA were well below this threshold, supporting that either of the two methods could be used for wear assessment (Figure 4).

### 4.2. Precision Measurements for CT‐RSA and RSA

The precision of proximal head penetration measured by CT‐RSA and RSA in our study, calculated as the 95% RI, was similar. We also found comparable precision to previous RSA studies for liner wear [17, 18]. To date, the only study to have assessed the precision of CT‐RSA for wear measurements was conducted in an experimental setting using a cadaveric model by Polus et al., who reported a precision of < 0.1 mm in all axes [6]. Our precision of 0.15 mm was slightly lower than that reported by Polus et al. However, a majority of studies on liner wear have not seen an increased risk for osteolysis or other complications if wear does not exceed 0.2 mm per year [14, 16] which implies that, with a better precision than 0.2 mm, we still find patients at risk for later revision.

### 4.3. Comparison CT‐RSA and RSA Method Efficacy

An advantage with CT‐RSA compared to conventional RSA for wear measurements is the possibility to use all metallic structures available in the cup, such as the thread around the cup. Thus, one does not have to rely on inserted tantalum beads or bone markers. Since tantalum beads can become obscured or migrate, the metallic thread around the cup provides a more stable reference for comparing proximal migration of the head. Current RSA research studies suggest exclusion rates due to marker occlusion ranging from 5% to 20% of the cohort [4, 19, 20]. Another advantage is the widespread accessibility to CT scanners, as CT‐RSA does not require specialized laboratories. CT scans can therefore be conducted at any hospital with the standard radiological personnel, in contrast to RSA, which demands specialized laboratories and trained staff for image capture.

Furthermore, CT scans are more sensitive for detection of early signs of loosening, such as osteolysis, progressive radiolucent lines around the implant, and early and continuous migration of the implant [5, 21]. Standard radiographs underestimate the presence of progressive radiolucent lines compared to CT scans [22], and CT‐RSA could therefore be a better method than RSA for detecting early radiological signs of loosening but also simultaneously provide information on bone quality. This could justify the slightly higher effective radiation dose of the CT‐RSA method.

In our study the first author received dedicated training at Sectra (developer of the CTMA software used in this study, Linköping, Sweden) for 2 days. Measurements were then performed by the first author and separately by an experienced observer using a standardized protocol. To assess reproducibility, inter‐observer agreement was evaluated using Bland–Altman analysis, which demonstrated good agreement between observers, despite differing levels of experience, suggesting that the method is reproducible across users with varying expertise.

### 4.4. Effective Radiation Dose

According to the Radiation Protection Act [23], patient doses of 1–10 mSv are permissible, while CT‐RSA guidelines recommend limiting each examination to 1–2 mSv, as studies often require 2–8 scans per patient [12]. Our low‐dose CT protocol achieves an effective dose of 0.2 mSv per scan, well below these limits and lower than the estimated 0.4 mSv dose of a standard AP pelvic radiograph [24].

The Exceed ABT (Zimmer Biomet) cemented polyethylene cup generally provides good visualization of the tantalum thread. However, in some patients, particularly one, segmentation was challenging due to the low‐dose CT protocol. A moderate dose increase would enhance segmentation, shorten CT‐RSA analysis time, and still remain within safe research limits.

### 4.5. Limitations

A first limitation is the small sample size used. To partly mitigate the effects of a small sample size, a *T* score was used instead of a *Z* score, which increases the reference intervals of our precision results. Nevertheless, the small sample size limited the statistical power of the study. Given our cohort size and the pooled SD of 0.2 mm, the minimum detectable difference between examination methods was 0.3 mm given conventional *ɑ* = 0.05 and 80% power. This, of course, affects the generalizability of our wear measurements. However, the primary aim of this study was to evaluate the feasibility of CT‐RSA for wear measurements rather than the wear measurements themselves.

The precision for RSA was determined in the original RCT [7] using double measurements conducted on a subset of patients from the cohort and using a 99% reference interval. As a result, we lacked the corresponding RSA double measurements for the patients included in this study. RSA precision was recalculated using the available repeat examinations from the original RCT. Consequently, direct comparison of precision between the two methods is limited. The precision ratio of 0.94 suggests similar precision between the two methods, but the wide bootstrap confidence interval indicates substantial uncertainty, which is expected given the limited number of double examinations. However, the primary aim of this study was the evaluation of agreement between the methods.

CT‐RSA relies more on user‐dependent assessment rather than objective metrics such as ME and CN used in conventional RSA to determine whether images are suitable for analysis. This may represent a limitation, particularly for less experienced users, as image selection and analysis could be influenced by the operator’s level of expertise.

One of the cups lacked the metal thread surrounding the cup, and therefore, tantalum beads were used instead. However, tantalum beads have been used for RSA measurements with high precision, supporting that this did not affect our results.

## 5. Conclusion

This exploratory study is the first study to compare CT‐RSA with conventional RSA for in vivo wear measurements after THA. At 6 years postoperatively, CT‐RSA demonstrated wear measurements comparable to conventional RSA. Bland–Altman plots showed good agreement between the methods. These findings suggest that CT‐RSA may be a viable alternative to conventional RSA for assessing polyethylene wear after THA. However, further validation in larger cohorts is needed.

## Funding

No funding was received specifically for this research project. However, the original study was supported by these foundations: the Åke Wiberg Foundation, Loo and Hans Ostermans Foundation, Sven Norén Foundation, and the regional agreement on medical training and clinical research (ALF) between Stockholm County Council and Karolinska Institute. Biomet partially funded the RSA examinations in the original study but had no further input or participation in the trial.

## Conflicts of Interest

Anders Brüggemann is president of the Swedish Hip and Knee Society and member of the steering committee for the Swedish Arthroplasty Register.

Olof Sköldenberg is Scientific Secretary for the Swedish Ethical Review Authority and Scientific Secretary of the Swedish Orthopedic Association.

Olof Sandberg is a full‐time employee at Sectra.

Nils P. Hailer has an unpaid leadership role in the Swedish National Board of Health and Welfare, Swedish Arthroplasty Register, Nordic Association of Arthroplasty Registers (NARA) and Swedish Research Council Infrastructure Biobank Sweden.

Cyrus Brodén does consultancy work for Johnson and Johnson.

The authors declare no conflicts of interest that could have influenced this study, with the exception of Olof Sandberg, who is an employee of Sectra.

## Data Availability

The data that support the findings for this study are available to other researchers upon request from the corresponding author.
